# Hierarchical network meta-analysis models for synthesis of evidence from randomised and non-randomised studies

**DOI:** 10.1186/s12874-023-01925-5

**Published:** 2023-04-22

**Authors:** Humaira Hussein, Keith R. Abrams, Laura J. Gray, Sumayya Anwer, Sofia Dias, Sylwia Bujkiewicz

**Affiliations:** 1 Biostatistics Research Group, Department of Population Health Sciences, University of Leicester, University Road, Leicester, LE1 7RH UK; 2 Department of Statistics, University of Warwick, Coventry, CV4 7AL UK; 3 Centre for Health Economics, University of York, York, YO10 5DD UK; 4 Centre for Reviews and Dissemination, University of York, York, YO10 5DD UK

**Keywords:** Real world evidence, Evidence synthesis, Hierarchical models, Bias adjustment

## Abstract

**Background:**

With the increased interest in the inclusion of non-randomised data in network meta-analyses (NMAs) of randomised controlled trials (RCTs), analysts need to consider the implications of the differences in study designs as such data can be prone to increased bias due to the lack of randomisation and unmeasured confounding. This study aims to explore and extend a number of NMA models that account for the differences in the study designs, assessing their impact on the effect estimates and uncertainty.

**Methods:**

Bayesian random-effects meta-analytic models, including naïve pooling and hierarchical models differentiating between the study designs, were extended to allow for the treatment class effect and accounting for bias, with further extensions allowing for bias terms to vary depending on the treatment class. Models were applied to an illustrative example in type 2 diabetes; using data from a systematic review of RCTs and non-randomised studies of two classes of glucose-lowering medications: sodium-glucose co-transporter 2 inhibitors and glucagon-like peptide-1 receptor agonists.

**Results:**

Across all methods, the estimated mean differences in glycated haemoglobin after 24 and 52 weeks remained similar with the inclusion of observational data. The uncertainty around these estimates reduced when conducting naïve pooling, compared to NMA of RCT data alone, and remained similar when applying hierarchical model allowing for class effect. However, the uncertainty around these effect estimates increased when fitting hierarchical models allowing for the differences in study design. The impact on uncertainty varied between treatments when applying the bias adjustment models. Hierarchical models and bias adjustment models all provided a better fit in comparison to the naïve-pooling method.

**Conclusions:**

Hierarchical and bias adjustment NMA models accounting for study design may be more appropriate when conducting a NMA of RCTs and observational studies. The degree of uncertainty around the effectiveness estimates varied depending on the method but use of hierarchical models accounting for the study design resulted in increased uncertainty. Inclusion of non-randomised data may, however, result in inferences that are more generalisable and the models accounting for the differences in the study design allow for more detailed and appropriate modelling of complex data, preventing overly optimistic conclusions.

**Supplementary Information:**

The online version contains supplementary material available at 10.1186/s12874-023-01925-5.

## Introduction

Network meta-analysis (NMA) is a widely used tool in health technology assessment (HTA) for the synthesis of direct and indirect evidence aiming to provide an overview of treatment effects [1]. Traditionally, NMAs have been carried out using data from randomised controlled trials (RCTs) as these have been considered the “gold-standard” for assessing effectiveness of interventions due to the randomisation techniques used and the strict criteria for inclusion/exclusion of individuals [2–4]. However, recently there has been an increased number of non-randomised observational and real-world studies conducted especially utilising large electronic health care databases. This has in turn highlighted an interest in including data from such studies in evidence synthesis, such as NMA, due to the epidemiological benefits they could provide [5]. However, such non-randomised data are considered to be inherently biased due to the lack of randomisation of individuals included and unmeasured confounding factors [1, 5]. If not accounted for, biased estimates from observational studies could in turn lead to biased estimates from the NMA, resulting in inappropriate conclusions drawn. Therefore, there is a growing need for methodological development and evaluation of methods for appropriate inclusion of non-randomised data in NMAs of RCTs and guidelines on such synthesis of data from randomised and non-randomised studies also begin to emerge [6].

Inclusion of non-randomised studies in evidence synthesis of RCT data has been considered for a number of reasons, typically either to allow for extension of evidence base when RCT data are sparse, looking to either improve the precision of the results or to bridge disconnected networks of RCT evidence, or to generalise the results to a broader population. A number of methods have been suggested to allow for inclusion of non-randomised data in NMAs of RCTs [5, 7–12]. Schmitz et al. [9] developed and compared a number of approaches, including naïve pooling, use of informative prior distributions and hierarchical models, by applying them to data in rheumatoid arthritis [9]. Schmitz et al. found that inclusion of observational evidence in NMA increased uncertainty of the pooled effectiveness estimates. Jenkins et al., who applied naïve pooling, a hierarchical model and power prior analysis to data in relapsing remitting multiple sclerosis, also obtained results with increased uncertainty compared to the analysis of RCT data alone, due to the increased between-study heterogeneity when incorporating data from non-randomised studies.

Bias, inherent in the observational data due to the lack of randomisation, has received a lot of consideration in the literature of methods for the analysis of individual participant data from observational studies [13]. The issue of bias in the meta-analysis of aggregate level data, including non-randomised comparative studies, has also been investigated, but not explored extensively in the context of real world evidence. Begg and Pilote proposed a model for adjusting for bias when including non-randomised evidence in meta-analysis; however, non-randomised data considered in this method were limited to single-arm studies [14]. In the context of NMA, a bias adjustment model for meta-analysis of comparative data has been introduced by Dias et al. [15]; in this case considering the risk of bias within RCTs. Schmitz et al. included bias adjustment in their hierarchical model, adjusting for overestimation (or underestimation) in the observational studies using an additive random bias term applied to the mean, at the basic parameter level in NMA, or for over precision using a multiplicative factor applied to the variance [9]. Efthimiou et al. propose a design-adjusted evidence synthesis method which combines data from randomized and non-randomised studies after adjusting the treatment effect estimates form the non-randomised evidence [8]. The two above methods, by Schmitz et al. and Efthimiou et al., assume that only data from non-randomised sources are biased. Verde proposed a Bayesian mixture model for pairwise meta-analysis, allowing for the true treatment effects in the meta-analysis to be a mixture of biased and unbiased effects [10].

Whilst in this paper we did not intend to carry out a full review of the literature on combining RCT and non-RCT data, the aim of this study was to evaluate and extend a number of methods for inclusion of non-randomised data in a NMA of RCTs. The existing methods that we focussed on included naïve pooling, hierarchical models and bias adjustment models, discussed by Schmitz et al. [9]. We first explore the models which account for the hierarchy of the data in terms of the grouping of treatments within classes as well as considering the different designs of included studies (i.e., randomised and non-randomised). We then extend these hierarchical models to allow for the class effect in the hierarchical model of different study design. We also explore the hierarchical model with bias adjustment, introduced by Schmitz et al., allowing for the bias for the non-randomised studies to be introduced at the individual study level as a random effect and extend it to allow for the average bias to vary across treatment classes.

We applied the methods to an illustrative example in type 2 diabetes assessing the impact of treatments within two classes of glucose-lowering medications; sodium-glucose co-transporter 2 inhibitors (SGLT-2is) and glucagon-like peptide-1 receptor agonists (GLP-1RAs) [16]. We illustrate how the methods can be utilised to model data from studies of different designs in NMA in more detail, and to explore the impact the modelling assumptions have on effect estimates and uncertainty.

## Methods

### Illustrative example

To illustrate the methods, we used an example in type 2 diabetes medications. Data were obtained from a systematic literature review of RCTs assessing the efficacy and safety of treatments within two classes of glucose lowering medications, SGLT-2is and GLP-1Ras, in individuals with type 2 diabetes undertaken by Hussein et al. [16]. The literature search from the review was repeated to identify non-randomised comparative studies conducted within the time-frame of the original systematic review (before April 2019). The evidence base for the NMA was further extended by including aggregate level data from the analysis of data from patients with type 2 diabetes included in the Clinical Practice Research Datalink. Data on the treatment effects of the medications included the mean change in HbA1c (%) from baseline after 24 ($\pm$ 8 weeks) and 52 weeks ($\pm$ 8 weeks).

### Basic network meta-analysis

The basic NMA random-effects model is as follows. The mean change in HbA1c, ${y}_{ik}$, in trial $i$ and arm $k$ is assumed to be approximately normally distributed with standard error $s{e}_{ik}$ and mean ${\theta }_{ik}$:

1 $${y}_{ik}\sim N\left({\theta }_{ik}, s{e}_{ik}^{2}\right)$$

Following a generalised linear model approach, an identity link function was used to model the true treatment effects (i.e. true mean differences from baseline), ${\delta }_{i,jk}$, between treatments in arm $k$ and arm $j$ in trial $i$, which are assumed to follow a normal distribution:

2 $${\theta }_{ik}={\mu }_{ij}+{\delta }_{i,jk}{I}_{\left\{k\ne j\right\}}$$

where

3 $${\delta }_{i,jk}\sim N({d}_{jk},{\sigma }^{2})$$

and ${\mu }_{ij}$ are the baseline treatment effects in each study *i*. The NMA models follow the assumption of consistency, which means that all studies would estimate the same relative effects if they had included all the treatments. This is modelled by expressing the mean treatment differences in terms of, so called, basic parameters (the effects of each treatment relative to a reference treatment in the network coded as treatment 1), i.e. ${d}_{jk}={d}_{1k}-{d}_{1j.}$. The assumption implies that the direct comparisons (where evidence exist from head-to-head studies for a given contrast) are exchangeable with indirect comparisons obtained using the above consistency rule.

Multi-arm adjustments were considered to account for the consistency between treatment comparisons within the same trial and correlation between treatment effects in comparison to the baseline treatment [10].

Following a Bayesian framework, prior distributions were placed on the parameters of the model in Eqs. (1)–(3). For example, we chose a non-informative uniform prior distribution for the heterogeneity parameter $\sigma \sim Uniform\left(\mathrm{0,5}\right)$, a “vague” normal prior distribution for the basic parameters ${d}_{1k}\sim N\left(0, 1000\right)$ and the baseline effects ${\mu }_{ij}\sim N\left(0, 10000\right)$. The model was initially applied to RCT data and data from non-randomised studies separately.

#### Shared parameter model

The data on treatment effects in our illustrative example have been reported using different formats: as either change from baseline within treatment arms or difference in change from baseline between treatment arms. To allow for the synthesis of all the relevant data, reported in such different ways, a shared parameter model was used following Dias et al. [7]. In addition to the model (1)–(2), representing the within-study model for the effects reported within treatment arms, we model the relative effects from studies reporting treatment differences between the treatment arms *k* and *j* as

4 $${y}_{i,jk}\sim N\left({\delta }_{i,jk}, s{e}_{i,jk}^{2}\right)$$

The relative effects ${\delta }_{i,jk}$ represent the shared parameter between the models for the two data formats (see Eq. (2)). These true effects ${\delta }_{i,jk}$ are assumed exchangeable within treatment contrasts in the network as described in Eq. (3) for the basic NMA.

### Network meta-analysis models for inclusion of non-randomised data

#### Model A – naïve pooling

The above basic NMA model, described by Eqs. (1)–(4), was applied to both RCT and non-randomised data combined, with no adjustments made for different sources of data or classes of treatments within the network.

#### Model B1 – two-level hierarchical model (treatment vs class)

The second type of model to be fitted was a two-level hierarchical model with treatments nested within treatment classes; i.e. treatments were nested within either SGLT-2i, GLP-1RA or placebo classes [17]. The model allows for borrowing of information across treatments within each class when estimating pooled treatment effects for individual treatments, which are of primary interest. It also allows for estimating an average effect within each treatment class, which may also be of interest. Updating Eqs. (2) and (3) leads to the random-effects model, which reads:

5 $${\theta }_{ik}={\mu }_{ij}+{\delta }_{i,jk}^{*}{I}_{\left\{k\ne j\right\}}, {\delta }_{i,jk}^{*}\sim N\left({d}_{jk}^{*},{\sigma }^{2}\right)$$

where ${d}_{jk}^{*}={d}_{1k,C}-{d}_{1j,C}$. The class-specific basic parameters ${d}_{1k,C}$ are assumed exchangeable:

6 $${d}_{1k,C}\sim N\left({D}_{C}, {\sigma }_{C}^{2}\right)$$

where ${D}_{C}$ denotes the pooled treatment effect estimate for treatments in the class $C$ of the interventions, relative to the reference treatment, and with between-treatment class-specific standard deviation ${\sigma }_{C}^{2}$. As for the naïve pooling model A, this model was extended to the shared parameter model to account for the differences in the way outcomes were reported. Note that in our illustrative example, we assumed fixed effect for the placebo class, with a prior distribution for the basic parameter ${d}_{1 placebo}\sim N\left(\mathrm{0,1000}\right)$, due to the fact that this class included only a single treatment.

Prior distributions were placed on parameters on the model. Similarly as in Model A – naïve pooling, we chose the following prior distributions for the parameters in Eqs. (5) and (6): $\sigma \sim Uniform\left(\mathrm{0,5}\right)$, ${\sigma }_{C}\sim Uniform\left(\mathrm{0,5}\right)$, ${D}_{C}\sim N(\mathrm{0,1000})$.

#### Model B2 – two-level hierarchical model (treatment vs design)

The third model we considered was a two-level hierarchical model, modelling the between-study heterogeneity of treatment effects within and across each study design (i.e. RCT and non-randomised studies). The model allows for differentiating between treatment effects from studies of different designs when estimating pooled treatment effects for individual treatments, which are of primary interest, and it allows for estimation of these average effects for each type of study design individually, and also overall across all studies (whilst taking into account of the across-design heterogeneity). Following the methods by Schmitz et al. [9];

7 $${\theta }_{ik}={\mu }_{ij}+{\delta }_{i,jk}{I}_{\left\{k\ne j\right\}}, {\delta }_{i,jk}\sim N\left({d}_{jk}^{design},{\sigma }^{2}\right)$$

where ${d}_{jk}^{design}$ is the design specific average treatment effect of treatment $k$ versus treatment $j$, with the mean ${d}_{jk}^{design}={d}_{1k}^{design}-{d}_{1j}^{design}$. The design specific basic parameters follow a common distribution

$${d}_{1k}^{design=RCT}\sim N({D}_{1k},{\upsigma }_{D}^{2}) {\,and\,d}_{1k}^{design=OBS}\sim N({D}_{1k},{\upsigma }_{D}^{2})$$

The following prior distributions were placed on parameters: ${\sigma }_{D}\sim Uniform\left(\mathrm{0,5}\right)$ and ${D}_{1k}\sim N\left(0, 1000\right)$.

#### Model B3 – three-level hierarchical model

This model was developed to extend the above two-level models (B1 and B2), by allowing for an additional level in the random-effects hierarchical NMA model to estimate the heterogeneity within study designs as well as estimating the heterogeneity within treatment classes in the network. Therefore, adapting the above model (7), the three-level hierarchical NMA model is as follows:

$${d}_{1k}^{design=RCT}\sim N({D}_{1k,C},{\upsigma }_{D}^{2}) {\,and\, d}_{1k}^{design=OBS}\sim N({D}_{1k,C},{\upsigma }_{D}^{2})$$

to allow for the class specific mean effects, which, similar to Eq. (6), are assumed exchangeable within each treatment class

$${D}_{1k,C}\sim N\left({D}_{C},{\sigma }_{C}^{2}\right).$$

Prior distributions were placed on the design level standard deviation ${\sigma }_{D}$, the class-specific standard deviations ${\sigma }_{C}$ and the class-specific mean ${D}_{C}$ in the same way as in the above two models.

#### Model C1 – bias adjustment assuming same bias by class

Observational studies are assumed to have additional risk of bias due to the absence of randomisation and unmeasured confounding. The bias adjustment model allows for this by including an additional bias parameter, ${\beta }_{i}$ for observational studies [9]. By including this additional term, the NMA model takes the following form:

8 $${\theta }_{ik}={\mu }_{ij}+{\delta }_{i,jk}{I}_{\left\{k\ne j\right\}}+{\beta }_{i}{I}_{\left\{design\right\}}$$

where:

$${I}_{\left\{design\right\}}=\left\{\begin{array}{c}0\, if\, design\, of\, study\, i=RCT\\ 1\, if\, design\, of\, study\, i=OBS\end{array}\right.$$

The true effects ${\delta }_{i,jk}$ follow a common distribution within each treatment contrast as in (3). The bias terms ${\beta }_{i}$ for each study i are assumed to follow a common distribution;

$${\beta }_{i}\sim N\left(B,{\kappa }^{2}\right),$$

with mean B and standard deviation $\upkappa$, which governs the extent to which different non-randomised studies vary in terms of the level of bias assumed. Non-informative prior distributions were placed on the following parameters: $\sigma \sim Uniform\left(\mathrm{0,5}\right)$, ${d}_{1k}\sim N(0, 1000)$, $B\sim N\left(\mathrm{0,1000}\right)$ and $\kappa \sim Uniform\left(\mathrm{0,5}\right).$

#### Model C2 – bias adjustment assuming varying bias by class

The above bias adjustment model assumes exchangeable biases across all observational studies regardless of treatment class being compared; however, the level of bias may differ across classes. Therefore, the model in Eq. (8) was extended to allow for a different degree of bias depending on the treatment class; placebo/standard care, SGLT-2i or GLP-1RA, whilst assuming the variability of the biases across classes is the same:

9 $${\theta }_{ik}={\mu }_{ij}+{\delta }_{i,jk}{I}_{\left\{k\ne j\right\}}+{\beta }_{i,C}^{*}{I}_{\left\{design\right\}}$$

where ${\mathrm{I}}_{\left\{\mathrm{design}\right\}}$ is defined in the same way as in model (8), the true effects ${\delta }_{i,jk}$ follow a common distribution within each treatment contrast as in (3) and the biases are exchangeable within each treatment class;

$${\beta }_{i,C}^{*}\sim N\left({B}_{C},{\kappa }^{2}\right)$$

with ${B}_{C}$ denoting the pooled bias estimate for class C of the interventions. Prior distributions for the model parameters were selected as those utilised in Model C1; with ${B}_{C}\sim N\left(\mathrm{0,1000}\right)$. Note that the standard deviation $\kappa$ is constant across classes and the model could be further extended to assume the ${B}_{C}$ parameters are exchangeable across treatment classes rather than independent

### Model fit and assessments

All models were implemented in WinBUGS [Version 1.4.3] [18]. The results were based on 600,000 simulations to ensure convergence of models. The first 100,000 simulations, classified as the “burn-in”, were discarded, with the next 500,000 simulations saved on which all results are based. Convergence on models was assessed through visual inspection of the trace and history plots. Model fit was compared using deviance information criterion (DIC). DICs can be used in Bayesian analysis as a measure of model fit, with smaller values indicating a better fit [19]. Total residual deviance was also compared to the total number of independent data points in the dataset being analysed. The results were reported as mean difference (95% credible intervals [CrI]) for the treatment effects in comparison to the reference treatment (canagliflozin) and median standard deviations (SDs) with 95% CrIs. Note that placebo could not be selected as the reference treatment in the network as there were no non-randomised studies with the placebo and the structure of some of the models require a reference treatment that is common to both study designs.

## Results

### Systematic review and network structure

**Fig. 1:**
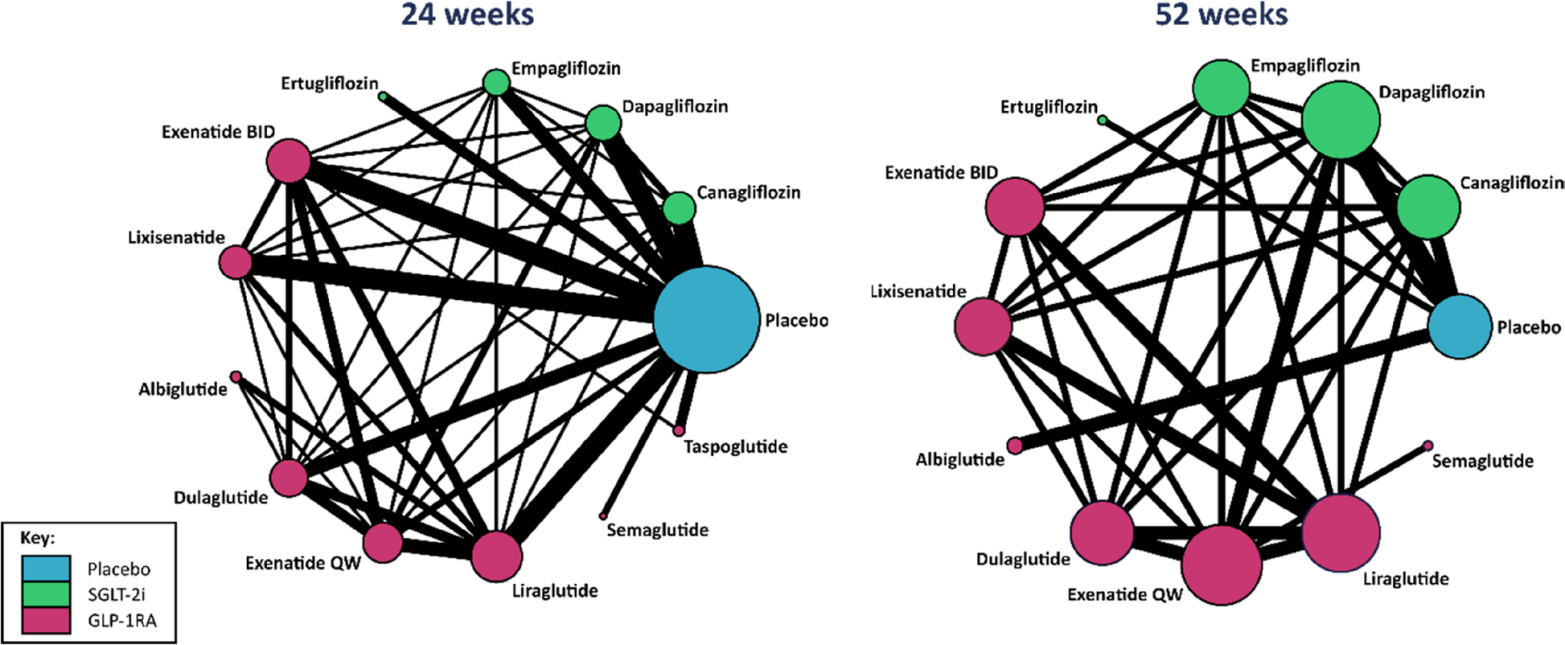
Network plots for the network meta-analysis of HbA1c (%) at 24 weeks and 52 weeks. Nodes represent treatments with sizes of the nodes proportional to the number of participants and the lines represent the direct comparisons between any two treatments with the width of the line proportional to the number of studies per contrast

In total, 74 studies were included in this NMA (study flow chart reported in Appendix 1 of the supplementary file); 64 papers were RCTs and 10 studies were non-randomised. Of the 64 RCTs included in the analysis, 53 reported outcomes at only 24 weeks, seven reported outcomes at only 52 weeks and four reported outcomes at both 24 and 52 weeks. Of the included observational studies, five reported outcomes at 24 weeks only, two reported outcomes at 52 weeks only and three reported outcomes at both 24 and 52 weeks (which included the aggregate data from the CPRD). Figure 1 displays the network structures. At 24 weeks, 13 unique treatments were compared: placebo, four treatments within the SGLT-2i class and eight treatments within the GLP-1RA class. The network was similar at 52 weeks, with the exception of taspoglutide being excluded due to no studies reporting outcomes at this time point for this treatment. The number of individuals with type 2 diabetes recruited to RCTs was on average 490 individuals (range: 50–2072 individuals) with observational studies on average studying larger populations (mean: 1863, range: 212–5141 individuals). List of references for the included studies is given in Additional file 1: Appendix 2 and the details of the studies, including the treatment arms, number of participants and the extracted data on treatment effects are reported in Appendix 3 of the supplementary file.

### Naïve pooling

**Fig. 2:**
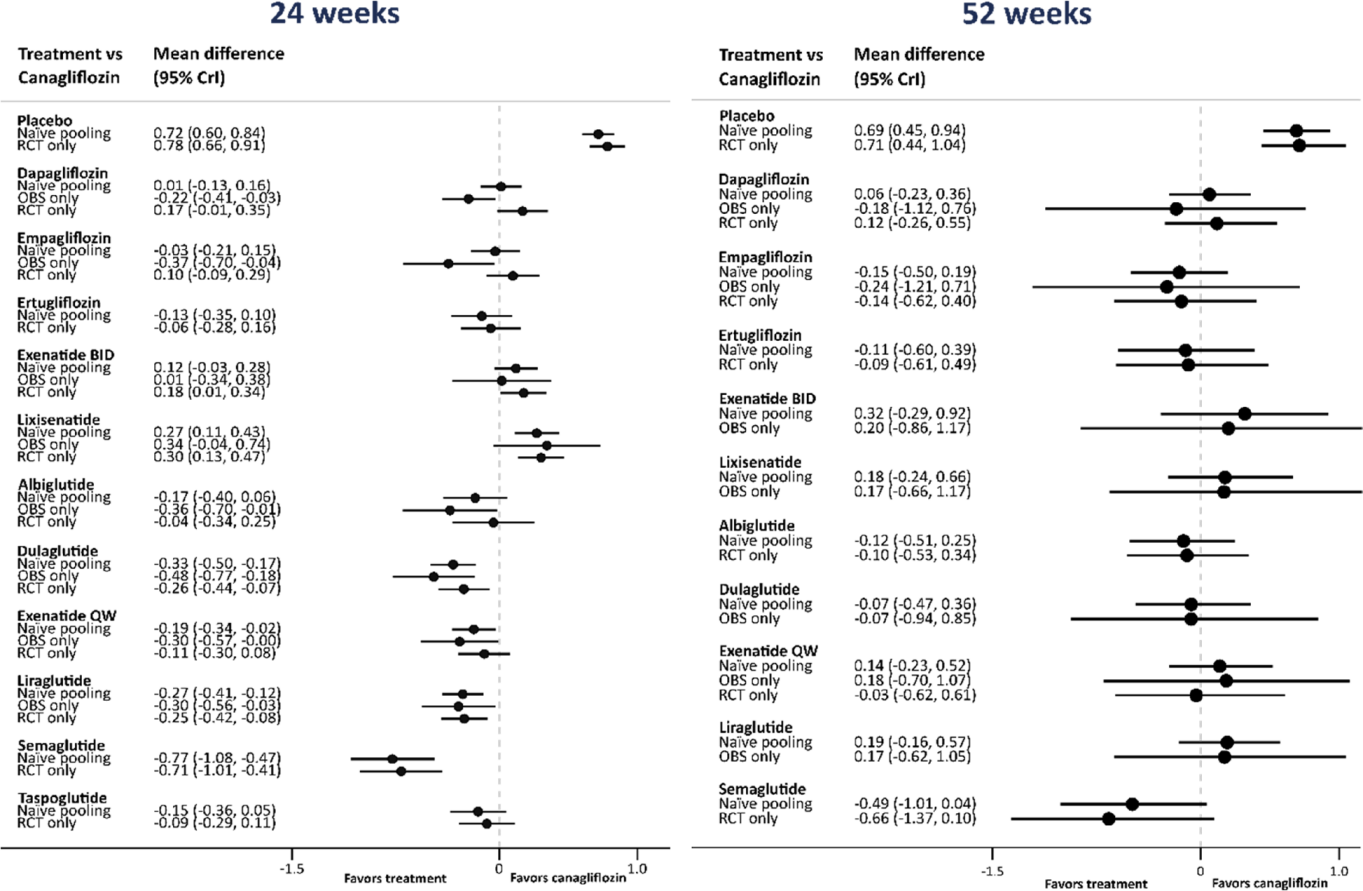
Network meta-analysis forest plots for analysis of HbA1c at 24 and 52 weeks using the naive pooling method. Note that for some of the treatments evidence was available only from either RCTs or from the non-randomised studies (denoted by OBS)

Figure 2, Table 1 (columns 2–4) and Table 2 (columns 2–4) report the mean differences (95% CrI), compared to the reference treatment canagliflozin, in change in HbA1c (%) from baseline after 24 and 52 weeks when analysing RCT data and observational data separately along with naïve pooling (Model A) method. Compared to placebo, the reference treatment canagliflozin reduced HbA1c by -0.72% (-0.84, -0.60) after 24 weeks and by -0.69% (-0.94, -0.45) after 52 weeks when using the naïve pooling method. There was no meaningful difference found between canagliflozin and other SGLT-2is (dapagliflozin, empagliflozin and ertugliflozin) at 24 weeks. However, most GLP-1RAs reduced HbA1c by a greater amount than canagliflozin, with the greatest reduction seen in semaglutide (-0.77% (-1.08, -0.47)). There were no other differences observed at 52 weeks. For treatment comparisons available from both RCTs and observational studies, the point estimates for the treatment effect obtained from the naïve pooling were typically between the mean effects obtained from the NMAs carried out separately for RCTs and for the non-randomised studies. Some of these effects obtained with reduced uncertainty compared to the estimates from the RCT data alone. This reduction in uncertainty, however, was relatively small. For example, at 24 weeks the effect of dapagliflozin relative to canagliflozin was 0.17 (-0.01, 0.35) from RCT data alone and 0.01 (-0.13, 0.16) from the naïve pooling of both sources of evidence, reducing the width of the 95% CrI by 19.4%. At 52 weeks the improvement in precision was much more pronounced due to the higher uncertainty around the effectiveness estimate for this endpoint (smaller number of studies). At 24 weeks, the effects of dapagliflozin and empagliflozin appeared to be in conflict when comparing the results across the two study designs (favouring the treatments compared to canagliflozin in the non-randomised studies but not in the RCTs). The estimated between-study heterogeneity differed across the two sets of data (RCTs and non-randomised studies), in particular at 52 weeks where it was obtained with much greater uncertainty.

**Table 1:** Mean difference from baseline ( 95% CrIs) for HbA1c (%) at 24 weeks for all models fitted vs reference treatment (canagliflozin) and median (95% CrIs) for the between-study standard deviation SD. Model A: naïve pooling, Model B1: two-level hierarchical model (treatment vs class), Model B2: two-level hierarchical model (treatment vs design), Model B3: three-level hierarchical model, Model C1: bias adjustment assuming the same bias by class, Model C2: bias adjustment allowing for bias to vary by class. ^a^SGLT-2i therapies (the remaining treatments, apart from the placebo, are from the GLP-1RA class)

**Treatment vs Canagliflozin**	**Model**							
	**RCT only**	**OBS only**	**Model A**	**Model B1**	**Model B2**	**Model B3**	**Model C1**	**Model C2**
**Effectiveness**								
**Dapagliflozin**^**a**^	0.17 (-0.01, 0.35)	-0.22 (-0.41, -0.03)	0.01 (-0.13, 0.16)	-0.00 (-0.14, 0.14)	0.00 (-0.24, 0.23)	-0.02 (-0.22, 0.19)	0.04 (-0.12, 0.22)	0.10 (-0.05, 0.25)
**Empagliflozin**^**a**^	0.10 (-0.09, 0.30)	-0.37 (-0.70, -0.04)	-0.03 (-0.21, 0.15)	-0.03 (-0.19, 0.13)	-0.05 (-0.34, 0.20)	-0.05 (-0.28, 0.16)	0.00 (-0.19, 0.19)	0.03 (-0.13, 0.19)
**Exenatide BID**	0.18 (0.01, 0.35)	0.01 (-0.34, 0.38)	0.12 (-0.04, 0.28)	0.11 (-0.04, 0.27)	0.13 (-0.13, 0.39)	0.09 (-0.16, 0.32)	0.13 (-0.02, 0.29)	0.12 (-0.02, 0.26)
**Lixisenatide**	0.30 (0.13, 0.47)	0.34 (-0.04, 0.74)	0.27 (0.11, 0.43)	0.26 (0.1, 0.42)	0.31 (0.06, 0.59)	0.24 (-0.06, 0.48)	0.27 (0.11, 0.43)	0.25 (0.11, 0.39)
**Albiglutide**	-0.04 (-0.34, 0.25)	-0.36 (-0.70, -0.01)	-0.17 (-0.40, 0.06)	-0.17 (-0.39, 0.05)	-0.16 (-0.45, 0.13)	-0.15 (-0.41, 0.10)	-0.14 (-0.38, 0.10)	-0.13 (-0.34, 0.07)
**Dulaglutide**	-0.26 (-0.44, -0.07)	-0.48 (-0.77, -0.18)	-0.33 (-0.50, -0.17)	-0.33 (-0.49, -0.17)	-0.32 (-0.58, -0.07)	-0.3 (-0.52, -0.06)	-0.31 (-0.48, -0.15)	-0.33 (-0.47, -0.18)
**Exenatide QW**	-0.11 (-0.30, 0.08)	-0.30 (-0.57, 0.00)	-0.19 (-0.34, -0.02)	-0.19 (-0.34, -0.03)	-0.16 (-0.41, 0.09)	-0.16 (-0.37, 0.06)	-0.17 (-0.33, 0.00)	-0.18 (-0.32, -0.03)
**Liraglutide**	-0.25 (-0.42, -0.08)	-0.30 (-0.56, -0.03)	-0.27 (-0.41, -0.12)	-0.26 (-0.41, -0.12)	-0.24 (-0.48, 0.00)	-0.23 (-0.44, 0.01)	-0.28 (-0.43, -0.12)	-0.31 (-0.45, -0.18)
**Placebo**	0.78 (0.66, 0.91)	-	0.72 (0.60, 0.84)	0.72 (0.61, 0.84)	0.74 (0.44, 1.06)	0.74 (0.47, 1.08)	0.73 (0.61, 0.85)	0.73 (0.63, 0.83)
**Ertugliflozin**^**a**^	-0.06 (-0.28, 0.16)	-	-0.13 (-0.35, 0.09)	-0.08 (-0.3, 0.1)	-0.10 (-0.44, 0.25)	-0.07 (-0.34, 0.18)	-0.11 (-0.33, 0.10)	-0.11 (-0.30, 0.08)
**Semaglutide**	-0.71 (-1.01, -0.41)	-	-0.77 (-1.08, -0.47)	-0.67 (-0.97, -0.37)	-0.75 (-1.14, -0.35)	-0.58 (-0.95, -0.14)	-0.76 (-1.06, -0.46)	-0.76 (-1.04, -0.48)
**Taspoglutide**	-0.09 (-0.29, 0.11)	-	-0.15 (-0.36, 0.05)	-0.15 (-0.35, 0.04)	-0.13 (-0.46, 0.22)	-0.13 (-0.4, 0.14)	-0.14 (-0.34, 0.06)	-0.14 (-0.32, 0.04)
**Class-level effects and bias**								
**D. SGLT-2i**				-0.04 (-0.55, 0.46)		-0.05 (-0.66, 0.55)		
**D. GLP-1RA**				-0.17 (-0.48, 0.12)		-0.15 (-0.45, 0.14)		
**Bias**							0.06 (-0.09, 0.2)	
**Bias. SGLT-2i**								-0.24 (-0.43, -0.03)
**Bias. GLP-1RA**								0.13 (0.00, 0.25)
**Heterogeneity**								
**SD**	0.1 (0.04, 0.16)	0.04 (0.00, 0.17)	0.11 (0.07, 0.16)	0.11 (0.07, 0.16)	0.09 (0.03, 0.14)	0.09 (0.02, 0.14)	0.1 (0.05, 0.16)	0.08 (0.01, 0.13)
**SD. SGLT-2i**				0.1 (0.0, 1.78)		0.12 (0.0, 2.03)		
**SD. GLP-1RA**				0.33 (0.18, 0.7)		0.31 (0.12, 0.69)		
**SD.design**					0.11 (0.02, 0.28)	0.1 (0.02, 0.26)		
**SD.bias**							0.09 (0.0, 0.26)	0.04 (0.0, 0.15)

**Table 2:** Mean difference from baseline ( 95% CrIs) for HbA1c (%) at 52 weeks for all models fitted vs reference treatment (canagliflozin) and median (95% CrIs) for the between-study standard deviation SD. Model A: naïve pooling, Model B1: two-level hierarchical model (treatment vs class), Model B2: two-level hierarchical model (treatment vs design), Model B3: three-level hierarchical model, Model C1: bias adjustment assuming the same bias by class, Model C2: bias adjustment allowing for bias to vary by class. ^a^SGLT-2i therapies (the remaining treatments, apart from the placebo, are from the GLP-1RA class)

**Treatment vs Canagliflozin**	**Model**							
	**RCT only**	**OBS only**	**Model A**	**Model B1**	**Model B2**	**Model B3**	**Model C1**	**Model C2**
**Effectiveness**								
**Dapagliflozin**^**a**^	0.12 (-0.26, 0.55)	-0.18 (-1.12, 0.76)	0.06 (-0.23, 0.37)	0.03 (-0.27, 0.33)	0.01 (-0.72, 0.68)	-0.02 (-0.39, 0.34)	0.10 (-0.18, 0.40)	0.06 (-0.22, 0.34)
**Empagliflozin**^**a**^	-0.14 (-0.62, 0.40)	-0.24 (-1.21, 0.50)	-0.15 (-0.51, 0.20)	-0.12 (-0.47, 0.21)	-0.17 (-0.90, 0.53)	-0.14 (-0.53, 0.24)	-0.15 (-0.48, 0.20)	-0.15 (-0.45, 0.17)
**Exenatide BID**	-	0.20 (-0.86, 0.53)	0.32 (-0.28, 0.90)	0.14 (-0.32, 0.62)	0.27 (-0.84, 1.30)	0.08 (-0.4, 0.6)	0.49 (-0.46, 1.30)	0.14 (-0.80, 1.07)
**Lixisenatide**	-	0.17 (-0.66, 0.48)	0.18 (-0.24, 0.67)	0.1 (-0.28, 0.62)	0.14 (-0.89, 1.14)	0.04 (-0.4, 0.5)	0.24 (-0.40, 0.88)	-0.11 (-0.82, 0.66)
**Dulaglutide**	-	-0.07 (-0.94, 0.46)	-0.07 (-0.47, 0.36)	-0.05 (-0.42, 0.34)	-0.07 (-1.07, 0.93)	-0.06 (-0.49, 0.37)	0.05 (-0.66, 0.66)	-0.20 (-0.91, 0.48)
**Exenatide QW**	-0.03 (-0.61, 0.60)	0.18 (-0.70, 0.46)	0.14 (-0.23, 0.52)	0.14 (-0.42, 0.34)	0.10 (-0.64, 0.81)	0.08 (-0.32, 0.47)	0.10 (-0.30, 0.51)	-0.09 (-0.54, 0.37)
**Liraglutide**	-	0.17 (-0.62, 0.43)	0.19 (-0.16, 0.57)	0.14 (-0.21, 0.49)	0.18 (-0.83, 1.15)	0.07 (-0.36, 0.5)	0.08 (-0.49, 0.72)	-0.25 (-0.89, 0.47)
**Placebo**	0.71 (0.44, 1.04)	-	0.69 (0.45, 0.94)	0.71 (0.47, 0.96)	0.70 (-0.23, 1.66)	0.72 (0.28, 1.17)	0.70 (0.49, 0.94)	0.69 (0.49, 0.90)
**Ertugliflozin**^**a**^	-0.09 (-0.61, 0.48)	-	-0.11 (-0.60, 0.40)	-0.07 (-0.5, 0.35)	-0.10 (-1.10, 0.92)	-0.08 (-0.55, 0.39)	-0.10 (-0.53, 0.36)	-0.11 (-0.52, 0.31)
**Albiglutide**	-0.10 (-0.53, 0.34)	-	-0.12 (-0.52, 0.25)	-0.07 (-0.4, 0.31)	-0.11 (-1.07, 0.87)	-0.06 (-0.44, 0.35)	-0.11 (-0.45, 0.23)	-0.12 (-0.44, 0.19)
**Semaglutide**	-0.66 (-1.36, 0.09)	-	-0.49 (-1.01, 0.05)	-0.29 (-0.76, 0.26)	-0.59 (-1.69, 0.42)	-0.25 (-0.8, 0.28)	-0.54 (-1.03, 0.00)	-0.72 (-1.26, 0.18)
**Class-level effects and bias**								
**d. SGLT-2i**				-0.05 (-0.99, 0.85)		-0.08 (-1.05, 0.87)		
**d. GLP-1RA**				0.02 (-0.36, 0.41)		-0.01 (-0.4, 0.39)		
**Bias**							0.21 (-0.5, 0.8)	
**Bias. SGLT-2i**								-0.21 (-1.06, 0.61)
**Bias. GLP-1RA**								0.26 (-0.34, 0.78)
**Heterogeneity**								
**SD**	0.10 (0.01, 0.5)	0.27 (0.02, 1.04)	0.13 (0.01, 0.33)	0.14 (0.01, 0.33)	0.11 (0.01, 0.32)	0.11 (0.01, 0.3)	0.09 (0.00, 0.3)	0.08 (0.00, 0.28)
**SD. SGLT-2i**				0.23 (0.01, 2.7)		0.23 (0.01, 2.81)		
**SD. GLP-1RA**				0.23 (0.03, 0.64)		0.2 (0.01, 0.64)		
**SD.design**					0.21 (0.01, 1.38)	0.14 (0.01, 0.39)a		
**SD.bias**							0.19 (0.01, 0.62)	0.14 (0.01, 0.57)

### Hierarchical models

Estimated overall mean differences in change from baseline in HbA1c (%), compared to the reference treatment of canagliflozin, when utilising the hierarchical models are reported in Table 1 (columns 5–7) for 24 weeks follow up time and Table 2 (columns 5–7) for 52 weeks follow up time. In comparison with the naïve-pooling method, hierarchical models provided similar effect estimates. The hierarchical model accounting for treatment classes (Model B1) resulted in the effect estimates with credible intervals similar to those obtained from the naïve-pooling model. However, the credible intervals were wider for the estimates from the hierarchical models allowing for differences in the study design (i.e. Model B2 and B3). For example, when considering the effect estimate for dapagliflozin in comparison to canagliflozin, the mean difference between the two treatments was close to 0 in all models. However, the estimate was obtained with a greater level of uncertainty in the two-level hierarchical model accounting for study design (0.00% (-0.24, 0.23)) and three-level hierarchical model (-0.02% (-0.27, 0.21)) in comparison to the two-level hierarchical model accounting for treatment classes (-0.01% (-0.15, 0.14)).

In all hierarchical models at 24 weeks, semaglutide showed the greatest reduction in HbA1c (%) in comparison to canagliflozin with a reduction of -0.71% (-1.00, -0.42) when using a two-level hierarchical model accounting for treatment classes and -0.75% reduction (-1.14, -0.35) when using a two-level hierarchical model accounting for study design. However, this reduction was smaller when using a three-level hierarchical model (-0.62% (-0.98, -0.12)) with increased uncertainty. This is likely to be due to the fact that the three-level hierarchical model takes into account the differences *between* treatments *within* classes of SGLT-2is and GLP-1RAs as well as the differences between study designs, allowing for additional variability.

### Bias-adjustment models

When considering the bias adjustment models, Model C1 assumes the same level of bias for all treatments in observational studies, regardless of treatment class. In this case, the effect estimates were similar to those obtained from the naïve pooling method. Further, these effect estimates had narrower credible intervals compared to those from the hierarchical models accounting for study design, as shown in Table 1 for the effect at 24 weeks and in Table 2 for the effect at 52 weeks. The bias term in this model was estimated to be 0.06 (-0.09, 0.20) at 24 weeks which suggests there was no substantial systematic discrepancy between RCT and observational studies (Table 1). However, when relaxing the assumption of the fully exchangeable bias and allowing the bias to vary across treatment classes (Model C2), the bias for SGLT-2is is estimated as -0.24 (-0.43, -0.03) and for GLP-1RAs as 0.13 (0.00, 0.25) at 24 weeks (Table 1). This suggests observational studies overestimated the effect of SGLT-2is by 0.24%, while GLP-1RAs were underestimated by 0.13%. While bias estimates differed across the two models, effect estimates and 95% credible intervals were similar (Tables 1 and 2), but slightly shifted in the direction of the bias. As observed in all other models, semaglutide showed the greatest reduction in HbA1c at 24 weeks in both bias-adjusted models (Model C1: -0.76% (-1.06, -0.46), Model C2: -0.76% (-1.04, -0.48)) (Table 1). There were no differences found at 52 weeks (Table 2); however, some of the estimates were obtained with reduced uncertainty compared to those from naïve pooling. For example, the effect of empagliflozin relative to canagliflozin at 52 weeks was -0.15 (-0.45, 0.17) from Model C2 and -0.15 (-0.51, 0.20) from naïve pooling.

### Model assessments

Table 3 reports the DIC, residual difference and the total number of independent data points for each NMA model at 24 and 52 weeks. At 24 and 52 weeks, the naïve-pooling model had the poorest fit (24 week DIC: -161.78, 52 week DIC: -15.15) in comparison to the hierarchical and bias-adjustment models fitted. At 24 weeks, the bias adjusted model assuming varying bias by class (Model C2) provided the best fit to the data according to the DIC (DIC: -175.68). However, the model showed poorest fit according to the residual deviance of 149.5 (compared to 137 data points) at 24 weeks. Both bias adjustment models had relatively large residual deviance at 52 weeks. The hierarchical models accounting for study design provided a good fit to the data in terms of both the DIC and residual deviance (two-level hierarchical model DIC: -174.26, three-level hierarchical model DIC: -173.81). At 52 weeks, the three-level hierarchical model accounting for treatment design and class provided the best fit to the data (DIC: -31.31), with the bias adjusted model varying the bias within classes also providing a good fit (DIC: -30.38). Hierarchical models accounting for the differences in the study design gave slightly lower between-study heterogeneity compared to the naïve pooling and the two level model with class effect, which, along with their good fit, suggests that these models may be preferred.

**Table 3:** Measures of model fit for models including both RCT and non-randomised studies. Model A: naïve pooling, Model B1: two-level hierarchical model (treatment vs class), Model B2: two-level hierarchical model (treatment vs design), Model B3: three-level hierarchical model, Model C1: bias adjustment assuming same bias by class, Model C2: bias adjustment allowing for bias to vary by class

**Model**	**DIC**		**Residual deviance**		**Total number of independent data points**	
	**24 weeks**	**52 weeks**	**24 weeks**	**52 weeks**	**24 weeks**	**52 weeks**
Model A	-161.78	-15.15	134.3	39.65	137	38
Model B1	-172.94	-29.81	135.6	39.74		
Model B2	-174.26	-28.96	135.8	38.93		
Model B3	-173.81	-31.31	136.3	39.92		
Model C1	-171.15	-29.68	138.3	46.56		
Model C2	-175.68	-30.38	149.5	45.71		

## Discussion

The methods used in this study provide a basis for inclusion of aggregate data from comparative non-randomised studies in a systematic review and NMA of RCTs. A number of methods were explored and developed further, which included naïve pooling, hierarchical models accounting for the design of the studies and bias adjustment for observational studies. All methods were applied to an illustrative example in type 2 diabetes medications.

In this systematic review and NMA of RCTs and non-randomised studies, a total of 64 RCTs and 10 observational studies were analysed. In most cases, the direction of effect was similar in both RCT data and non-randomised data, which is supported by current research [20]. However, in contrast to the RCTs, the observational studies favoured two SGLT-2i therapies, compared to the reference treatment. Naïve-pooling averaged the effect estimate between what was observed in RCTs and non-randomised studies, with most effect estimates having similar or smaller credible intervals in comparison to the results of NMA of RCT data alone.

In order to account for the limitations of non-randomised studies, hierarchical models and bias adjusted models were explored. In this study, hierarchical models fitted accounted for the design of the study, which was further extended to consider the classification of treatments within the SGLT-2i and GLP-1RA class. As in previous studies [9, 21], effect estimates were similar to those from the naïve pooling method but credible intervals were often wider. In particular, allowing for additional heterogeneity across studies of different designs increased credible intervals. By allowing for additional levels of heterogeneity, the impact of the over-precision of the estimates from the observational studies on the pooled effects may be reduced.

Bias adjusted models, applied to data from our example of type 2 diabetes, resulted in similar effect estimates, if slightly shifted to the direction of the bias, compared to the naïve pooling model. Similar to Dias et al. [15], between trial heterogeneity decreased when adjusting for bias, thus suggesting some of this heterogeneity was explained by the bias in observational studies. Interestingly, allowing bias to vary by class, relaxing the assumption that bias could be in the same direction regardless of treatment, models provided a better fit to the data according to DIC. This suggests that the magnitude and directionality of bias could differ by class and it may not be appropriate to assume the same bias for all observational studies.

### Limitations

There are a number of limitations that need to be considered in this study. Firstly, this study has considered a single dataset and illustrative example. While this is a relatively large NMA, considering a number of treatments and studies, it included a relatively small number of non-randomised studies, which may have contributed excessively to the increased level of uncertainty. It is important to consider the effect of these models in alternative datasets, which may depend on a number of factors. Previously published studies showed similar effects as this study when utilising the naïve pooling model and hierarchical model accounting for study design [9, 21]. The results from this study are promising but would need further investigation to understand the implications in other datasets. Future studies should also consider using simulation to assess the performance of these methods under a range a scenarios. Secondly, the non-randomised studies included in the NMA on average contributed a larger proportion of individuals compared to RCTs. This could potentially lead to the increased impact of the non-randomised studies on the pooled effectiveness estimates, which is a limitation particularly in the presence of unmeasured confounding. Thirdly, the issue of double-counting of individuals in NMAs including observational studies was not considered in this study. As the number of real-world and observational studies using large electronic health care databases increase, it is likely that individuals could be included multiple times in evidence synthesis due to the same database being used or individuals included in the databases also taking part in RCTs, thus artificially inflating precision [22]. However, allowing for further heterogeneity across study designs and by introducing a bias factor, may mitigate the impact of this issue due to the allowance for increased uncertainty. Fourth, bias within RCTs was not considered in this NMA. Risk of bias assessment was completed in the original systematic review and NMA for RCTs. Most studies showed low risk of bias in RCTs and so adjusting for bias in RCTs in this case may have minimal impact but further work could consider adjusting for bias within RCTs as well as observational studies by, for example, adapting a Bayesian mixture hierarchical model proposed by Verde [10] to an NMA and allowing for bias to vary according to treatment class. In fact, an extension of the model by Verde to NMA was recently proposed by Hamza et al., who also make software available to analysts [23]. Finally, this systematic review and NMA only considered aggregate level data for both RCTs and observational studies. It would be important to consider the extension of these methods when including IPD for both RCTs and observational studies, as recently proposed by Hamza et al. [23]. Moreover, further work could also consider the impact of the quality of the effectiveness estimates from the non-randomised studies when modelling bias. For example, there is likely heterogeneity in the way treatment effects are estimated and reported. Some studies may use appropriate methods of adjustment for confounding whereas others may not. Such information could also be used when deciding how to share the bias parameters; across the studies providing better quality estimates vs those of poorer quality.

## Conclusions

The inclusion of observational data in NMAs of RCTs is gaining considerable traction in HTA due to the many benefits such as increasing evidence base, potentially connecting disconnected networks and allowing for more generalizable inferences. Methods such as hierarchical NMA and bias adjustment allow for more detailed modelling of the heterogeneity between study designs and can also be extended to allow for differences between treatment classes or account for differences in treatment doses. Both, hierarchical and bias adjustment models can provide a better fit to the data in comparison to naïve pooling and should be explored when conducting evidence synthesis. While the methods developed may ameliorate the effects of overestimation in observational studies, further analysis such as simulation studies would need to be conducted to investigate the capabilities of these models.

## Supplementary Information

**Additional file 1: Appendix 1.** PRISMA flow chart of included randomised controlled trials and observational studies in the network meta-analysis. **Appendix 2.** List of references of the studies included in the network meta-analysis. **Appendix 3.** Table of data extracted from studies included in the network meta-analysis.

## Data Availability

All data generated or analysed during this study are included in this published article (and its supplementary information files).
