# Supplementary material for: Hierarchical network meta-analysis models for synthesis of evidence from randomised and non-randomised studies
Source: BMC Med Res Methodol. 2023 Apr 22;23:97. doi: 10.1186/s12874-023-01925-5 (PMC10122363; doi:10.1186/s12874-023-01925-5)
Supplement: Supplementary file 1 — Additional file 1: Appendix 1. PRISMA flow chart of included randomised controlled trials and observational studies in the network meta-analysis. Appendix 2. List of references of the studies included in the network meta-analysis. Appendix 3. Table of data extracted from studies included in the network meta-analysis. [file 12874_2023_1925_MOESM1_ESM.docx]

# Supplementary material

## Appendix 1

PRISMA flow chart of included randomised controlled trials and observational studies in the network meta-analysis


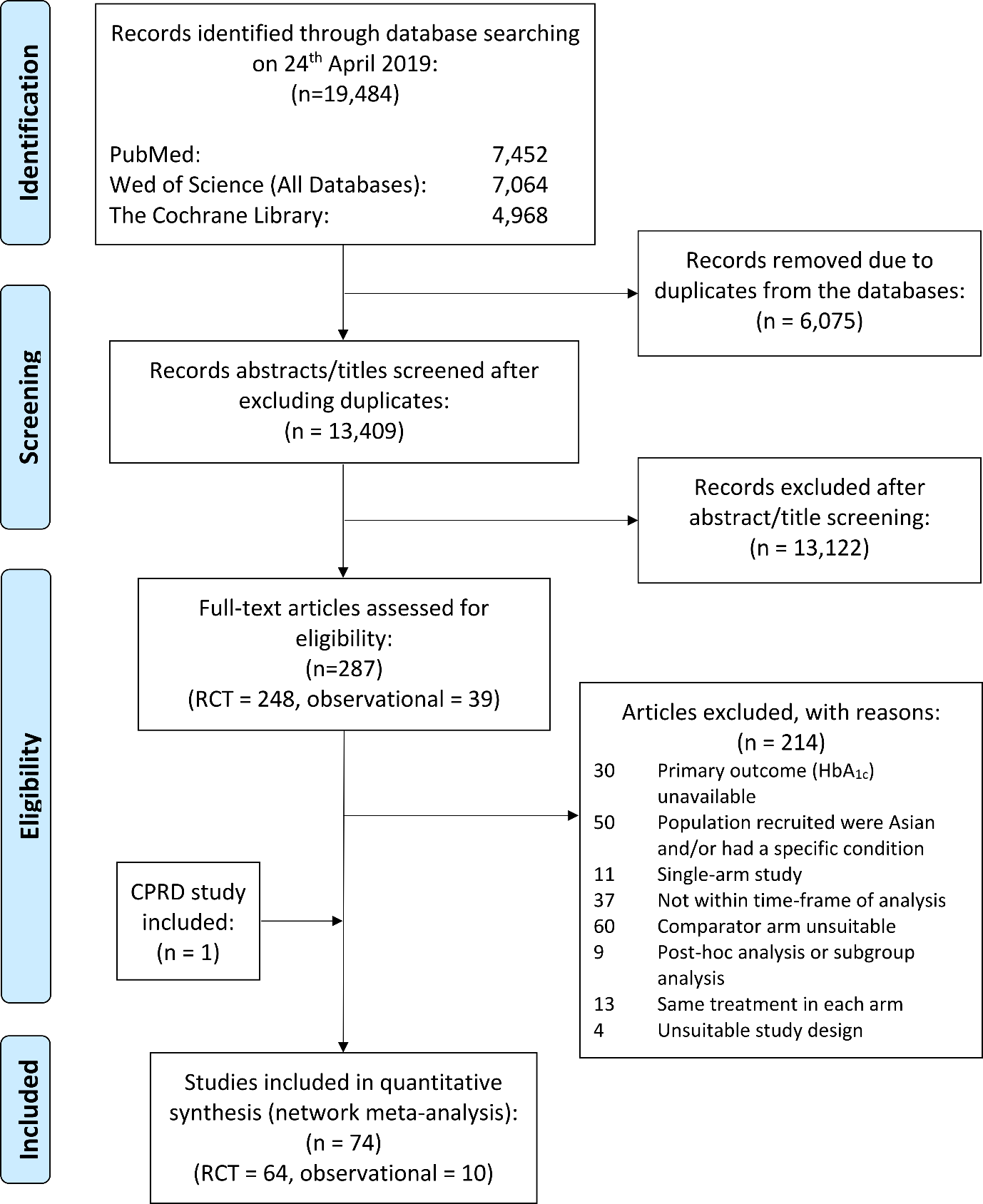


## Appendix 2

The table below includes the list of references of the studies included in the network meta-analysis. Reference index for each study is used to link the reference with study details presented in Appendix 3 below. Study type is reported as either randomised controlled trial (RCT) or observational study (OBS).

| **Reference index** | **Study type** | **Reference** |
| --- | --- | --- |
| **24 weeks and 52 weeks (+- 8 weeks)** | | |
| A1 | RCT | Ahmann, A., Rodbard, H. W., Rosenstock, J., Lahtela, J. T., Loredo, L., Tornøe, K., Boopalan, A. and Nauck, M. A. (2015) Efficacy and safety of liraglutide versus placebo added to basal insulin analogues (with or without metformin) in patients with type 2 diabetes: a randomized, placebo-controlled trial. *Diabetes, Obesity and Metabolism,* *17*(11), pp. 1056-1064. |
| A2 | RCT | Ahren, B., Leguizamo Dimas, A., Miossec, P., Saubadu, S. and Aronson, R. (2013) Efficacy and safety of lixisenatide once-daily morning or evening injections in type 2 diabetes inadequately controlled on metformin (GetGoal-M). *Diabetes Care,* *36*(9), pp. 2543-50. |
| A3 | RCT | Bailey, C. J., Gross, J. L., Pieters, A., Bastien, A. and List, J. F. (2010) Effect of dapagliflozin in patients with type 2 diabetes who have inadequate glycaemic control with metformin: a randomised, double-blind, placebo-controlled trial. *The Lancet,* *375*(9733), pp. 2223-33. |
| A4 | RCT | Bailey, C. J., Iqbal, N., T'Joen, C. and List, J. F. (2012) Dapagliflozin monotherapy in drug-naive patients with diabetes: a randomized-controlled trial of low-dose range. *Diabetes, Obesity and Metabolism,* *14*(10), pp. 951-9. |
| A5 | RCT | Blevins, T., Pullman, J., Malloy, J., Yan, P., Taylor, K., Schulteis, C., Trautmann, M. and Porter, L. (2011) DURATION-5: exenatide once-weekly resulted in greater improvements in glycemic control compared with exenatide twice daily in patients with type 2 diabetes. *The Journal of Clinical Endocrinology & Metabolism,* *96*(5), pp. 1301-10. |
| A6 | RCT | Bode, B., Stenlof, K., Sullivan, D., Fung, A. and Usiskin, K. (2013) Efficacy and safety of canagliflozin treatment in older subjects with type 2 diabetes mellitus: a randomized trial. *Hospital Practice,* *41*(2), pp. 72-84. |
| A7 | RCT | Bolli, G. B., Munteanu, M., Dotsenko, S., Niemoeller, E., Boka, G., Wu, Y. and Hanefeld, M. (2014) Efficacy and safety of lixisenatide once daily vs. placebo in people with type 2 diabetes insufficiently controlled on metformin (GetGoal-F1). *Diabetic Medicine,* *31*(2), pp. 176-84. |
| A8 | RCT | Buse, J. B., Henry, R. R., Han, J., Kim, D. D., Fineman, M. S. and Baron, A. D. (2004) Effects of exenatide (exendin-4) on glycemic control over 30 weeks in sulfonylurea-treated patients with type 2 diabetes. *Diabetes Care,* *27*(11), pp. 2628-35. |
| A9 | RCT | Buse, J. B., Bergenstal, R. M., Glass, L. C., Heilmann, C. R., Lewis, M. S., Kwan, A. Y., Hoogwerf, B. J. and Rosenstock, J. (2011) Use of twice-daily exenatide in basal insulin-treated patients with type 2 diabetes: a randomized, controlled trial. *Annals of Internal Medicine,* *154*(2), pp. 103-12. |
| A10 | RCT | Buse, J. B., Nauck, M., Forst, T., Sheu, W. H., Shenouda, S. K., Heilmann, C. R., Hoogwerf, B. J., Gao, A., Boardman, M. K., Fineman, M., Porter, L. and Schernthaner, G. (2013) Exenatide once weekly versus liraglutide once daily in patients with type 2 diabetes (DURATION-6): a randomised, open-label study. *The Lancet,* *381*(9861), pp. 117-24. |
| A11 | RCT | Buse, J. B., Rosenstock, J., Sesti, G., Schmidt, W. E., Montanya, E., Brett, J. H., Zychma, M. and Blonde, L. (2009) Liraglutide once a day versus exenatide twice a day for type 2 diabetes: a 26-week randomised, parallel-group, multinational, open-label trial (LEAD-6). *The Lancet,* *374*(9683), pp. 39-47. |
| A12 | RCT | Dagogo-Jack, S., Liu, J., Eldor, R., Amorin, G., Johnson, J., Hille, D., Liao, Y., Huyck, S., Golm, G., Terra, S. G., Mancuso, J. P., Engel, S. S. and Lauring, B. (2017) Efficacy and safety of the addition of ertugliflozin in patients with type 2 diabetes mellitus inadequately controlled with metformin and sitagliptin: the VERTIS SITA2 placebo-controlled randomized study. *Diabetes, Obesity and Metabolism,* *20*(3), pp. 530-540. |
| A13 | RCT | DeFronzo, R. A., Ratner, R. E., Han, J., Kim, D. D., Fineman, M. S. and Baron, A. D. (2005) Effects of exenatide (exendin-4) on glycemic control and weight over 30 weeks in metformin-treated patients with type 2 diabetes. *Diabetes Care,* *28*(5), pp. 1092-100. |
| A14 | RCT | Drucker, D. J., Buse, J. B., Taylor, K., Kendall, D. M., Trautmann, M., Zhuang, D. and Porter, L. (2008) Exenatide once-weekly versus twice daily for the treatment of type 2 diabetes: a randomised, open-label, non-inferiority study. *Lancet,* *372*(9645), pp. 1240-50. |
| A15 | RCT | Dungan, K. M., Tofe Povedano, S., Forst, T., Gonzalez Gonzalez, J. G., Atisso, C., Sealls, W. and Fahrbach, J. L. (2014) Once-weekly dulaglutide versus once-daily liraglutide in metformin-treated patients with type 2 diabetes (AWARD-6): a randomised, open-label, phase 3, non-inferiority trial. *The Lancet,* *384*(9951), pp. 1349-1357. |
| A16 | RCT | Dungan, K. M., Weitgasser, R., Perez Manghi, F., Pintilei, E., Fahrbach, J. L., Jiang, H. H., Shell, J. and Robertson, K. E. (2016) A 24-week study to evaluate the efficacy and safety of once-weekly dulaglutide added on to glimepiride in type 2 diabetes (AWARD-8). *Diabetes, Obesity and Metabolism,* *18*(5), pp. 475-82. |
| A17 | RCT | Ferrannini, E., Jimenez Ramos, S., Salsali, A., Tang, W. and List, J. F. (2010) Dapagliflozin monotherapy in type 2 diabetic patients with inadequate glycemic control by diet and exercise: a randomized, double-blind, placebo-controlled, phase 3 trial. *Diabetes Care,* *33*(10), pp. 2217-2224. |
| A18 | RCT | Forst, T., Guthrie, R., Goldenberg, R., Yee, J., Vijapurkar, U., Meininger, G. and Stein, P. (2014) Efficacy and safety of canagliflozin over 52 weeks in patients with type 2 diabetes on background metformin and pioglitazone. *Diabetes, Obesity and Metabolism*, *16*(5), pp. 467-477. |
| A19 | RCT | Frías, J. P., Guja, C., Hardy, E., Ahmed, A., Dong, F., Öhman, P. and Jabbour, S. A. (2016) Exenatide once weekly plus dapagliflozin once daily versus exenatide or dapagliflozin alone in patients with type 2 diabetes inadequately controlled with metformin monotherapy (DURATION-8): a 28 week, multicentre, double-blind, phase 3, randomised controlled trial. *The lancet Diabetes & endocrinology*, *4*(12), pp. 1004-1016. |
| A20 | RCT | Gadde, K. M., Vetter, M. L., Iqbal, N., Hardy, E. and Ohman, P. (2017) Efficacy and safety of autoinjected exenatide once-weekly suspension versus sitagliptin or placebo with metformin in patients with type 2 diabetes: the DURATION-NEO-2 randomized clinical study. *Diabetes, Obesity and Metabolism,* *19*(7), pp. 979-988. |
| A21 | RCT | Guja, C., Frias, J. P., Somogyi, A., Jabbour, S., Wang, H., Hardy, E. and Rosenstock, J. (2018) Effect of exenatide QW or placebo, both added to titrated insulin glargine, in uncontrolled type 2 diabetes: the DURATION-7 randomized study. *Diabetes, Obesity and Metabolism*. |
| A22 | RCT | Henry, R. R., Mudaliar, S., Kanitra, L., Woloschak, M. and Balena, R. (2012) Efficacy and safety of taspoglutide in patients with type 2 diabetes inadequately controlled with metformin plus pioglitazone over 24 weeks: T-Emerge 3 trial. *The Journal of Clinical Endocrinology & Metabolism*, *97*(7), pp. 2370-2379. |
| A23 | RCT | Hollander, P., Lasko, B., Barnett, A. H., Bengus, M., Kanitra, L., Pi-Sunyer, F. X. and Balena, R. (2013) Effects of taspoglutide on glycemic control and body weight in obese patients with type 2 diabetes (T-emerge 7 study). *Obesity*, *21*(2), pp. 238-247. |
| A24 | RCT | Häring, H. U., Merker, L., Seewaldt-Becker, E., Weimer, M., Meinicke, T., Woerle, H. J. and Broedl, U. C. (2013) Empagliflozin as add-on to metformin plus sulfonylurea in patients with type 2 diabetes: a 24-week, randomized, double-blind, placebo-controlled trial. *Diabetes Care,* *36*(11), pp. 3396-404. |
| A25 | RCT | Kovacs, C. S., Seshiah, V., Swallow, R., Jones, R., Rattunde, H., Woerle, H. J. and Broedl, U. C. (2014) Empagliflozin improves glycaemic and weight control as add-on therapy to pioglitazone or pioglitazone plus metformin in patients with type 2 diabetes: a 24-week, randomized, placebo-controlled trial. *Diabetes, Obesity and Metabolism*, *16*(2), pp. 147-158. |
| A26 | RCT | Lavalle-González, F. J., Januszewicz, A., Davidson, J., Tong, C., Qiu, R., Canovatchel, W. and Meininger, G. (2013) Efficacy and safety of canagliflozin compared with placebo and sitagliptin in patients with type 2 diabetes on background metformin monotherapy: a randomised trial. *Diabetologia*, *56*(12), pp. 2582-2592. |
| A27 | RCT | Lind, M., Hirsch, I. B., Tuomilehto, J., Dahlqvist, S., Ahren, B., Torffvit, O., Attvall, S., Ekelund, M., Filipsson, K., Tengmark, B. O., Sjoberg, S. and Pehrsson, N. G. (2015) Liraglutide in people treated for type 2 diabetes with multiple daily insulin injections: randomised clinical trial (MDI Liraglutide trial). *BMJ,* *351*, pp. h5364. |
| A28 | RCT | Lingvay, I., Desouza, C. V., Lalic, K. S., Rose, L., Hansen, T., Zacho, J. and Pieber, T. R. (2018) A 26-week randomized controlled trial of semaglutide once daily versus liraglutide and placebo in patients with type 2 diabetes suboptimally controlled on diet and exercise with or without metformin. *Diabetes Care,* *41*(9), pp. 1926‐1937. |
| A29 | RCT | Liutkus, J., Rosas Guzman, J., Norwood, P., Pop, L., Northrup, J., Cao, D. and Trautmann, M. (2010) A placebo-controlled trial of exenatide twice-daily added to thiazolidinediones alone or in combination with metformin. *Diabetes, Obesity and Metabolism,* *12*(12), pp. 1058-65. |
| A30 | RCT | Ludvik, B., Frias, J. P., Tinahones, F. J., Wainstein, J., Jiang, H., Robertson, K. E., Garcia-Perez, L. E., Woodward, D. B. and Milicevic, Z. (2018) Dulaglutide as add-on therapy to SGLT2 inhibitors in patients with inadequately controlled type 2 diabetes (AWARD-10): a 24-week, randomised, double-blind, placebo-controlled trial. *The lancet Diabetes & endocrinology,* *6*(5), pp. 370-381. |
| A31 | RCT | Matthaei, S., Bowering, K., Rohwedder, K., Grohl, A. and Parikh, S. (2015) Dapagliflozin improves glycemic control and reduces body weight as add-on therapy to metformin plus sulfonylurea: a 24-week randomized, double-blind clinical trial. *Diabetes Care,* *38*(3), pp. 365-72. |
| A32 | RCT | Moretto, T. J., Milton, D. R., Ridge, T. D., Macconell, L. A., Okerson, T., Wolka, A. M. and Brodows, R. G. (2008) Efficacy and tolerability of exenatide monotherapy over 24 weeks in antidiabetic drug-naive patients with type 2 diabetes: a randomized, double-blind, placebo-controlled, parallel-group study. *Clinical Therapeutics,* *30*(8), pp. 1448-60. |
| A33 | RCT | Nauck, M., Weinstock, R. S., Umpierrez, G. E., Guerci, B., Skrivanek, Z. and Milicevic, Z. (2014) Efficacy and safety of dulaglutide versus sitagliptin after 52 weeks in type 2 diabetes in a randomized controlled trial (AWARD-5). *Diabetes Care*, *37*(8), pp. 2149-2158. |
| A34 | RCT | Nauck, M., Rizzo, M., Johnson, A., Bosch-Traberg, H., Madsen, J. and Cariou, B. (2016) Once-daily liraglutide versus lixisenatide as add-on to metformin in type 2 diabetes: a 26-week randomized controlled clinical trial. *Diabetes Care*, *39*(9), pp. 1501-1509. |
| A35 | RCT | Pinget, M., Goldenberg, R., Niemoeller, E., Muehlen-Bartmer, I., Guo, H. and Aronson, R. (2013) Efficacy and safety of lixisenatide once daily versus placebo in type 2 diabetes insufficiently controlled on pioglitazone (GetGoal-P). *Diabetes, Obesity and Metabolism,* *15*(11), pp. 1000-7. |
| A36 | RCT | Pratley, R. E., Nauck, M. A., Barnett, A. H., Feinglos, M. N., Ovalle, F., Harman-Boehm, I., Ye, J., Scott, R., Johnson, S., Stewart, M. and Rosenstock, J. (2014) Once-weekly albiglutide versus once-daily liraglutide in patients with type 2 diabetes inadequately controlled on oral drugs (HARMONY 7): a randomised, open-label, multicentre, non-inferiority phase 3 study. *The lancet Diabetes & endocrinology,* *2*(4), pp. 289-97. |
| A37 | RCT | Raz, I., Fonseca, V., Kipnes, M., Durrwell, L., Hoekstra, J., Boldrin, M. and Balena, R. (2012) Efficacy and safety of taspoglutide monotherapy in drug-naive type 2 diabetic patients after 24 weeks of treatment: results of a randomized, double-blind, placebo-controlled phase 3 study (T-emerge 1). *Diabetes Care,* *35*(3), pp. 485-7. |
| A38 | RCT | Riddle, M. C., Forst, T., Aronson, R., Sauque-Reyna, L., Souhami, E., Silvestre, L., Ping, L. and Rosenstock, J. (2013) Adding once-daily lixisenatide for type 2 diabetes inadequately controlled with newly initiated and continuously titrated basal insulin glargine: a 24-week, randomized, placebo-controlled study (GetGoal-Duo 1). *Diabetes Care,* *36*(9), pp. 2497-503. |
| A39 | RCT | Riddle, M. C., Aronson, R., Home, P., Marre, M., Niemoeller, E., Miossec, P., Ping, L., Ye, J. and Rosenstock, J. (2013) Adding once-daily lixisenatide for type 2 diabetes inadequately controlled by established basal insulin: a 24-week, randomized, placebo-controlled comparison (GetGoal-L). *Diabetes Care,* *36*(9), pp. 2489-96. |
| A40 | RCT | Rodbard, H. W., Lingvay, I., Reed, J., de la Rosa, R., Rose, L., Sugimoto, D., Araki, E., Chu, P. L., Wijayasinghe, N. and Norwood, P. (2018) Semaglutide added to basal insulin in type 2 diabetes (SUSTAIN 5): a randomized, controlled trial. *The Journal of Clinical Endocrinology & Metabolism,* *103*(6), pp. 2291-2301. |
| A41 | RCT | Rodbard, H. W., Seufert, J., Aggarwal, N., Cao, A., Fung, A., Pfeifer, M. and Alba, M. (2016) Efficacy and safety of titrated canagliflozin in patients with type 2 diabetes mellitus inadequately controlled on metformin and sitagliptin. *Diabetes, Obesity and Metabolism*, *18*(8), pp. 812-819. |
| A42 | RCT | Roden, M., Weng, J., Eilbracht, J., Delafont, B., Kim, G., Woerle, H. J. and Broedl, U. C. (2013) Empagliflozin monotherapy with sitagliptin as an active comparator in patients with type 2 diabetes: a randomised, double-blind, placebo-controlled, phase 3 trial. *The lancet Diabetes & endocrinology,* *1*(3), pp. 208-19. |
| A43 | RCT | Rosenstock, J., Vico, M., Wei, L., Salsali, A. and List, J. F. (2012) Effects of dapagliflozin, an SGLT2 inhibitor, on HbA(1c), body weight, and hypoglycemia risk in patients with type 2 diabetes inadequately controlled on pioglitazone monotherapy. *Diabetes Care*, *35*(7), pp. 1473-1478. |
| A44 | RCT | Rosenstock, J., Frias, J., Pall, D., Charbonnel, B., Pascu, R., Saur, D., Darekar, A., Huyck, S., Shi, H., Lauring, B. and Terra, S. G. (2018) Effect of ertugliflozin on glucose control, body weight, blood pressure and bone density in type 2 diabetes mellitus inadequately controlled on metformin monotherapy (VERTIS MET). *Diabetes Obesity & Metabolism,* *20*(3), pp. 520-529. |
| A45 | RCT | Rosenstock, J., Hanefeld, M., Shamanna, P., Min, K. W., Boka, G., Miossec, P., Zhou, T., Muehlen-Bartmer, I. and Ratner, R. E. (2014) Beneficial effects of once-daily lixisenatide on overall and postprandial glycemic levels without significant excess of hypoglycemia in type 2 diabetes inadequately controlled on a sulfonylurea with or without metformin (GetGoal-S). *Journal of Diabetes and Its Complications,* *28*(3), pp. 386-92. |
| A46 | RCT | Rosenstock, J., Balas, B., Charbonnel, B., Bolli, G. B., Boldrin, M., Ratner, R. and Balena, R. (2013) The fate of taspoglutide, a weekly GLP-1 receptor agonist, versus twice-daily exenatide for type 2 diabetes: the T-emerge 2 trial. *Diabetes Care,* *36*(3), pp. 498-504. |
| A47 | RCT | Rosenstock, J., Raccah, D., Korányi, L., Maffei, L., Boka, G., Miossec, P. and Gerich, J. E. (2013) Efficacy and safety of lixisenatide once daily versus exenatide twice daily in type 2 diabetes inadequately controlled on metformin: a 24-week, randomized, open-label, active-controlled study (GetGoal-X). *Diabetes Care*, *36*(10), pp. 2945-2951. |
| A48 | RCT | Russell-Jones, D., Vaag, A., Schmitz, O., Sethi, B. K., Lalic, N., Antic, S., Zdravkovic, M., Ravn, G. M. and Simo, R. (2009) Liraglutide vs insulin glargine and placebo in combination with metformin and sulfonylurea therapy in type 2 diabetes mellitus (LEAD-5 met+SU): a randomised controlled trial. *Diabetologia,* *52*(10), pp. 2046-55. |
| A49 | RCT | Softeland, E., Meier, J. J., Vangen, B., Toorawa, R., Maldonado-Lutomirsky, M. and Broedl, U. C. (2017) Empagliflozin as add-on therapy in patients with type 2 diabetes inadequately controlled with linagliptin and metformin: a 24-week randomized, double-blind, parallel-group trial. *Diabetes Care,* *40*(2), pp. 201-209. |
| A50 | RCT | Sorli, C., Harashima, S. I., Tsoukas, G. M., Unger, J., Karsbol, J. D., Hansen, T. and Bain, S. C. (2017) Efficacy and safety of once-weekly semaglutide monotherapy versus placebo in patients with type 2 diabetes (SUSTAIN 1): a double-blind, randomised, placebo-controlled, parallel-group, multinational, multicentre phase 3a trial. *The lancet Diabetes & endocrinology,* *5*(4), pp. 251-260. |
| A51 | RCT | Stenlöf, K., Cefalu, W. T., Kim, K. A., Alba, M., Usiskin, K., Tong, C., Canovatchel, W. and Meininger, G. (2013) Efficacy and safety of canagliflozin monotherapy in subjects with type 2 diabetes mellitus inadequately controlled with diet and exercise. *Diabetes, Obesity and Metabolism*, *15*(4), pp. 372-382. |
| A52 | RCT | Terra, S. G., Focht, K., Davies, M., Frias, J., Derosa, G., Darekar, A., Golm, G., Johnson, J., Saur, D., Lauring, B. and Dagogo-Jack, S. (2017) Phase III, efficacy and safety study of ertugliflozin monotherapy in people with type 2 diabetes mellitus inadequately controlled with diet and exercise alone. *Diabetes, Obesity and Metabolism,* *19*(5), pp. 721-728. |
| A53 | RCT | Vanderheiden, A., Harrison, L., Warshauer, J., Li, X., Adams-Huet, B. and Lingvay, I. (2016) Effect of adding liraglutide vs placebo to a high-dose lnsulin regimen in patients with type 2 diabetes: a randomized clinical trial. *JAMA Internal Medicine,* *176*(7), pp. 939-47. |
| A54 | RCT | Wilding, J. P. H., Woo, V., Soler, N. G., Pahor, A., Sugg, J., Rohwedder, K., Parikh, S. and Dapagliflozin 006 Study, G. (2012) Long-term efficacy of dapagliflozin in patients with type 2 diabetes mellitus receiving high doses of insulin: a randomized trial. *Annals of Internal Medicine,* *156*(6), pp. 405-U50. |
| A55 | RCT | Wilding, J. P., Charpentier, G., Hollander, P., Gonzalez-Galvez, G., Mathieu, C., Vercruysse, F., Usiskin, K., Law, G., Black, S., Canovatchel, W. and Meininger, G. (2013) Efficacy and safety of canagliflozin in patients with type 2 diabetes mellitus inadequately controlled with metformin and sulphonylurea: a randomised trial. *International Journal of Clinical Practice,* *67*(12), pp. 1267-82. |
| A56 | RCT | Wit, H. M., Vervoort, G. M., Jansen, H. J., Grauw, W. J., Galan, B. E. and Tack, C. J. (2014) Liraglutide reverses pronounced insulin-associated weight gain, improves glycaemic control and decreases insulin dose in patients with type 2 diabetes: a 26 week, randomised clinical trial (ELEGANT). *Diabetologia*, *57*(9), pp. 1812-1819. |
| A57 | RCT | Wysham, C., Blevins, T., Arakaki, R., Colon, G., Garcia, P., Atisso, C., Kuhstoss, D. and Lakshmanan, M. (2014) Efficacy and safety of dulaglutide added onto pioglitazone and metformin versus exenatide in type 2 diabetes in a randomized controlled trial (AWARD-1). *Diabetes Care*, *37*(8), pp. 2159-2167. |
| B1 | OBS | Blonde, L., Patel, C., Bookhart, B., Pfeifer, M., Chen, Y.W. and Wu, B., 2018. A real-world analysis of glycemic control among patients with type 2 diabetes treated with canagliflozin versus dapagliflozin. Current medical research and opinion, 34(6), pp.1143-1152. |
| B2 | OBS | Buysman, E.K., Sikirica, M.V., Thayer, S.W., Bogart, M., DuCharme, M.C. and Joshi, A.V., 2018. Real-world comparison of treatment patterns and effectiveness of albiglutide and liraglutide. Journal of comparative effectiveness research, 7(2), pp.89-100. |
| B3 | OBS | Fadini, G.P., Bonora, B.M., Lapolla, A., Fattor, B., Morpurgo, P.S., Simioni, N. and Avogaro, A., 2019. Comparative effectiveness of exenatide once‐weekly versus liraglutide in routine clinical practice: A retrospective multicentre study and meta‐analysis of observational studies. Diabetes, obesity & metabolism, 21(5), p.1255. |
| B4 | OBS | Saunders, W.B., Nguyen, H. and Kalsekar, I., 2016. Real-world glycemic outcomes in patients with type 2 diabetes initiating exenatide once weekly and liraglutide once daily: a retrospective cohort study. Diabetes, metabolic syndrome and obesity: targets and therapy, 9, p.217. |
| B5 | OBS | Unni, S., Wittbrodt, E., Ma, J., Schauerhamer, M., Hurd, J., Ruiz‐Negrón, N. and McAdam‐Marx, C., 2018. Comparative effectiveness of once‐weekly glucagon‐like peptide‐1 receptor agonists with regard to 6‐month glycaemic control and weight outcomes in patients with type 2 diabetes. Diabetes, Obesity and Metabolism, 20(2), pp.468-473. |
| B6 | OBS | Jassim, Z., Elajez, R., Khudair, I., Al Anany, R. and Al-Adawi, R.M., 2019. Efficacy and safety of once daily liraglutide versus twice daily exenatide in type 2 diabetic patients in Qatar: an observational study. Journal of Pharmaceutical Health Services Research, 10(1), pp.73-80. |
| B7 | OBS | Mody, R., Huang, Q., Yu, M., Zhao, R., Patel, H., Grabner, M. and Landó, L.F., 2019. Adherence, persistence, glycaemic control and costs among patients with type 2 diabetes initiating dulaglutide compared with liraglutide or exenatide once weekly at 12‐month follow‐up in a real‐world setting in the United States. Diabetes, obesity & metabolism, 21(4), p.920. |
| **52 weeks (+- 8 weeks) only** | | |
| C1 | RCT | Ahmann, A. J., Capehorn, M., Charpentier, G., Dotta, F., Henkel, E., Lingvay, I., Holst, A. G., Annett, M. P. and Aroda, V. R. (2018) Efficacy and safety of once-weekly semaglutide versus exenatide ER in subjects with type 2 diabetes (SUSTAIN 3): a 56-week, open-label, randomized clinical trial. *Diabetes Care,* *41*(2), pp. 258-266. |
| C2 | RCT | Jabbour, S. A., Frias, J. P., Hardy, E., Ahmed, A., Wang, H., Ohman, P. and Guja, C. (2018) Safety and efficacy of exenatide once weekly plus dapagliflozin once daily versus exenatide or dapagliflozin alone in patients with type 2 diabetes inadequately controlled with metformin monotherapy: 52-week results of the DURATION-8 randomized controlled trial. *Diabetes Care,* *41*(10), pp. 2136-2146. |
| C3 | RCT | Matthaei, S., Bowering, K., Rohwedder, K., Sugg, J., Parikh, S. and Johnsson, E. (2015) Durability and tolerability of dapagliflozin over 52-weeks as add-on to metformin and sulphonylurea in type 2 diabetes. *Diabetes, Obesity and Metabolism*, *17*(11), pp. 1075-1084. |
| C4 | RCT | Nauck, M. A., Stewart, M. W., Perkins, C., Jones-Leone, A., Yang, F., Perry, C., Reinhardt, R. R. and Rendell, M. (2016) Efficacy and safety of once-weekly GLP-1 receptor agonist albiglutide (HARMONY 2): 52 week primary endpoint results from a randomised, placebo-controlled trial in patients with type 2 diabetes mellitus inadequately controlled with diet and exercise. *Diabetologia*, *59*(2), pp. 266-274. |
| C5 | RCT | Neal, B., Perkovic, V., de Zeeuw, D., Mahaffey, K. W., Fulcher, G., Ways, K., Desai, M., Shaw, W., Capuano, G., Alba, M., Jiang, J., Vercruysse, F., Meininger, G. and Matthews, D. (2015) Efficacy and safety of canagliflozin, an inhibitor of sodium-glucose cotransporter 2, when used in conjunction with insulin therapy in patients with type 2 diabetes. *Diabetes Care,* *38*(3), pp. 403-11. |
| C6 | RCT | Reusch, J., Stewart, M. W., Perkins, C. M., Cirkel, D. T., Ye, J., Perry, C. R., Reinhardt, R. R. and Bode, B. W. (2014) Efficacy and safety of once-weekly glucagon-like peptide 1 receptor agonist albiglutide (HARMONY 1 trial): 52-week primary endpoint results from a randomized, double-blind, placebo-controlled trial in patients with type 2 diabetes mellitus not controlled on pioglitazone, with or without metformin. *Diabetes, Obesity and Metabolism,* *16*(12), pp. 1257-64. |
| C7 | RCT | Roden, M., Merker, L., Christiansen, A. V., Roux, F., Salsali, A., Kim, G., Stella, P., Woerle, H. J. and Broedl, U. C. (2015) Safety, tolerability and effects on cardiometabolic risk factors of empagliflozin monotherapy in drug-naïve patients with type 2 diabetes: a double-blind extension of a phase III randomized controlled trial. *Cardiovascular Diabetology*, *14*, p. 154. |
| D1 | OBS | Feher, M., Vega-Hernandez, G., Mocevic, E., Buysse, B., Myland, M., Power, G.S., Husemoen, L.L.N., Kim, J. and Witte, D.R., 2017. Effectiveness of liraglutide and lixisenatide in the treatment of type 2 diabetes: real-world evidence from The Health Improvement Network (THIN) database in the United Kingdom. Diabetes therapy, 8(2), pp.417-431. |
| D1 | OBS | McAdam-Marx, C., Nguyen, H., Schauerhamer, M.B., Singhal, M., Unni, S., Ye, X. and Cobden, D., 2016. Glycemic control and weight outcomes for exenatide once weekly versus liraglutide in patients with type 2 diabetes: a 1-year retrospective cohort analysis. Clinical therapeutics, 38(12), pp.2642-2651. |
| *Abbreviations: OBS, Observational; RCT, Randomised Controlled Trial.*  *Note: Reference index is assigned to each included study to reference throughout the manuscript.* | | |

## Appendix 3

Table of data extracted from studies included in the network meta-analysis. The columns represent the following information:

1. Reference index refers to the study specific reference defined in Appendix 2
2. First author refers to first author surname of the published study
3. Study type reported as either randomised controlled trial (RCT) or observational study (OBS)
4. Treatment details medication used in each arm
5. N = Number of individuals randomised to each treatment arm
6. Glycated haemoglobin (HbA1c), measured as %, reported as either mean change from baseline (standard error) within the treatment arms or the difference in the mean change from baseline (standard error) between treatment arms, which is then used in the NMA models

| **Reference index** | **First Author** | **Study type** | **Treatment** | **N** | **HbA1c [%] (standard error)** |
| --- | --- | --- | --- | --- | --- |
| **24 weeks** | | | | | |
| A1 | Ahmann | RCT | PLA/SC | 225 | -0.11 (0.07) |
|  |  |  | LIR | 225 | -1.30 (0.07) |
| A2 | Ahren | RCT | PLA/SC | 170 | -0.40 (0.08) |
|  |  |  | LIX | 510 | -0.85 (0.10) |
| A3 | Bailey | RCT | PLA/SC | 265 | -0.30 (0.07) |
|  |  |  | DAPA | 134 | -0.77 (0.10) |
| A4 | Bailey | RCT | PLA/SC | 68 | 0.02 (0.12) |
|  |  |  | DAPA | 66 | -0.98 (0.17) |
| A5 | Blevins | RCT | ExBID | 123 | -0.90 (0.10) |
|  |  |  | ExQW | 129 | -1.60 (0.10) |
| A6 | Bode | RCT | PLA/SC | 232 | -0.03 (0.06) |
|  |  |  | CANA | 468 | -0.66 (0.09) |
| A7 | Bolli | RCT | PLA/SC | 158 | -0.42 (0.10) |
|  |  |  | LIX | 308 | -0.88 (0.14) |
| A8 | Buse | RCT | PLA/SC | 123 | 0.12 (0.09) |
|  |  |  | ExBID | 254 | -0.66 (0.16) |
| A9 | Buse | RCT | PLA/SC | 122 | -1.04 (0.09) |
|  |  |  | ExBID | 137 | -1.74 (0.09) |
| A10 | Buse | RCT | ExQW | 390 | -1.28 (0.05) |
|  |  |  | LIR | 386 | -1.48 (0.05) |
| A11 | Buse | RCT | ExBID | 231 | -0.79 (0.08) |
|  |  |  | LIR | 233 | -1.12 (0.08) |
| A12 | Dagogo | RCT | PLA/SC | 153 | -0.09 (0.05) |
|  |  |  | ERTU | 309 | -0.82 (0.10) |
| A13 | DeFronzo | RCT | PLA/SC | 113 | 0.10 (0.10) |
|  |  |  | ExBID | 223 | -0.60 (0.14) |
| A14 | Drucker | RCT | ExQW | 147 | -1.87 (0.08) |
|  |  |  | ExBID | 148 | -1.54 (0.08) |
| A15 | Dungan | RCT | DUL | 299 | -1.42 (0.05) |
|  |  |  | LIR | 300 | -1.36 (0.05) |
| A16 | Dungan | RCT | PLA/SC | 60 | -0.10 (0.14) |
|  |  |  | DUL | 239 | -1.40 (0.08) |
| A17 | Ferrannini | RCT | PLA/SC | 75 | -0.23 (0.10) |
|  |  |  | DAPA | 278 | -0.81 (0.11) |
| A18 | Forst | RCT | PLA/SC | 115 | -0.26 (0.07) |
|  |  |  | CANA | 227 | -0.96 (0.10) |
| A19 | Frias | RCT | DAPA | 230 | -1.39 (0.09) |
|  |  |  | ExQW | 227 | -1.60 (0.10) |
| A20 | Gadde | RCT | PLA/SC | 61 | -0.40 (0.19) |
|  |  |  | ExQW | 181 | -1.13 (0.11) |
| A21 | Guja | RCT | PLA/SC | 230 | -0.23 (0.08) |
|  |  |  | ExQW | 231 | -0.96 (0.08) |
| A22 | Henry | RCT | PLA/SC | 94 | -0.45 (0.10) |
|  |  |  | TAS | 219 | -1.38 (0.13) |
| A23 | Hollander | RCT | PLA/SC | 143 | -0.09 (0.06) |
|  |  |  | TAS | 149 | -0.81 (0.06) |
| A24 | Häring | RCT | PLA/SC | 225 | -0.17 (0.05) |
|  |  |  | EMPA | 441 | -0.80 (0.07) |
| A25 | Kovacs | RCT | PLA/SC | 165 | -0.11 (0.07) |
|  |  |  | EMPA | 333 | -0.66 (0.10) |
| A26 | Lavalle-González | RCT | PLA/SC | 183 | -0.17 (0.06) |
|  |  |  | CANA | 735 | -0.86 (0.06) |
| A27 | Lind | RCT | PLA/SC | 59 | -0.42 (0.12) |
|  |  |  | LIR | 63 | -1.54 (0.11) |
| A28 | Lingvay | RCT | PLA/SC | 129 | -0.05 (0.08) |
|  |  |  | LIR | 129 | -1.09 (0.16) |
| A29 | Liutkus | RCT | PLA/SC | 54 | -0.10 (0.23) |
|  |  |  | ExBID | 111 | -0.84 (0.20) |
| A30 | Ludvik | RCT | PLA/SC | 140 | -0.54 (0.06) |
|  |  |  | DUL | 283 | -1.28 (0.09) |
| A31 | Matthaei | RCT | PLA/SC | 108 | -0.17 (0.07) |
|  |  |  | DAPA | 108 | -0.86 (0.07) |
| A32 | Moretto | RCT | PLA/SC | 77 | -0.20 (0.10) |
|  |  |  | ExBID | 155 | -0.80 (0.14) |
| A33 | Nauck | RCT | PLA/SC | 177 | 0.03 (0.07) |
|  |  |  | DUL | 606 | -1.12 (0.08) |
| A34 | Nauck | RCT | LIX | 202 | -1.24 (0.07) |
|  |  |  | LIR | 202 | -1.81 (0.06) |
| A35 | Pinget | RCT | PLA/SC | 161 | -0.34 (0.10) |
|  |  |  | LIX | 323 | -0.90 (0.09) |
| A36 | Pratley | RCT | ALB | 404 | -0.78 (0.05) |
|  |  |  | LIR | 408 | -0.99 (0.05) |
| A37 | Raz | RCT | PLA/SC | 115 | -0.09 (0.07) |
|  |  |  | TAS | 239 | -1.10 (0.09) |
| A38 | Riddle | RCT | PLA/SC | 166 | -0.40 (0.10) |
|  |  |  | LIX | 327 | -0.70 (0.10) |
| A39 | Riddle | RCT | PLA/SC | 223 | -0.40 (0.10) |
|  |  |  | LIX | 223 | -0.70 (0.10) |
| A40 | Rodbard | RCT | PLA/SC | 133 | -0.10 (0.09) |
|  |  |  | SEM | 263 | -1.60 (0.13) |
| A41 | Rodbard | RCT | PLA/SC | 106 | -0.01 (0.12) |
|  |  |  | CANA | 107 | -0.91 (0.12) |
| A42 | Roden | RCT | PLA/SC | 228 | 0.08 (0.05) |
|  |  |  | EMPA | 448 | -0.72 (0.07) |
| A43 | Rosenstock | RCT | PLA/SC | 139 | -0.42 (0.08) |
|  |  |  | DAPA | 281 | -0.89 (0.11) |
| A44 | Rosenstock | RCT | PLA/SC | 209 | 0.00 (0.08) |
|  |  |  | ERTU | 412 | -0.80 (0.07) |
| A45 | Rosenstock | RCT | PLA/SC | 274 | -0.10 (0.07) |
|  |  |  | LIX | 554 | -0.85 (0.06) |
| A46 | Rosenstock | RCT | ExBID | 373 | -0.98 (0.08) |
|  |  |  | TAS | 776 | -1.28 (0.12) |
| A47 | Rosenstock | RCT | ExBID | 315 | -0.96 (0.05) |
|  |  |  | LIX | 315 | -0.79 (0.05) |
| A48 | Russell-Jones | RCT | PLA/SC | 114 | -0.24 (0.11) |
|  |  |  | LIR | 230 | -1.33 (0.09) |
| A49 | Softeland | RCT | PLA/SC | 88 | 0.14 (0.09) |
|  |  |  | EMPA | 200 | -0.61 (0.11) |
| A50 | Sorli | RCT | PLA/SC | 129 | -0.02 (0.10) |
|  |  |  | SEM | 258 | -1.50 (0.14) |
| A51 | Stenlöf | RCT | PLA/SC | 192 | 0.14 (0.07) |
|  |  |  | CANA | 392 | -0.90 (0.09) |
| A52 | Terra | RCT | PLA/SC | 153 | 0.20 (0.09) |
|  |  |  | ERTU | 307 | -0.87 (0.12) |
| A53 | Vanderheiden | RCT | PLA/SC | 36 | 0.00 (0.20) |
|  |  |  | LIR | 35 | -0.90 (0.23) |
| A54 | Wilding | RCT | PLA/SC | 150 | -0.13 (0.08) |
|  |  |  | CANA | 307 | -0.95 (0.11) |
| A55 | Wilding | RCT | PLA/SC | 193 | -0.39 (0.10) |
|  |  |  | DAPA | 405 | -0.92 (0.14) |
| A56 | Wit | RCT | PLA/SC | 24 | 0.01 (0.12) |
|  |  |  | LIR | 26 | -0.77 (0.11) |
| A57 | Wysham | RCT | PLA/SC | 141 | -0.46 (0.08) |
|  |  |  | ExBID | 276 | -0.99 (0.06) |
|  |  |  | DUL | 559 | -1.40 (0.09) |
| B1 | Blonde | OBS | CANA | 558 | Reference |
|  |  |  | DAPA | 558 | -0.26 (0.09) |
| B2 | Buysman | OBS | ALB | 244 | -1.00 (0.11) |
|  |  |  | LIR | 244 | -1.00 (0.10) |
| B3 | Fadini | OBS | ExQW | 204 | -0.70 (0.07) |
|  |  |  | LIR | 410 | -0.70 (0.05) |
| B4 | Saunders | OBS | ExQW | 664 | -0.64 (0.05) |
|  |  |  | LIR | 3283 | -0.65 (0.02) |
| B5 | Unni | OBS | ALB | 131 | -0.60 (0.13) |
|  |  |  | DUL | 201 | -0.60 (0.11) |
|  |  |  | ExQW | 2133 | -0.50 (0.03) |
| B6 | Jassim | OBS | ExBID | 114 | 0.10 (0.17) |
|  |  |  | LIR | 98 | -0.21 (0.18) |
| B7 | Mody | OBS | DUL | 420 | -1.10 (0.08) |
|  |  |  | LIR | 433 | -0.86 (0.08) |
| B7 | Mody | OBS | DUL | 307 | -1.15 (0.10) |
|  |  |  | ExQW | 296 | -0.92 (0.10) |
| NA | Hussein | OBS | CANA | 154 | -0.93 (0.16) |
|  |  |  | DAPA | 610 | -1.05 (0.08) |
|  |  |  | EMPA | 238 | -1.21 (0.12) |
|  |  |  | ExBID | 155 | -0.83 (0.16) |
|  |  |  | LIX | 182 | -0.50 (0.16) |
|  |  |  | DUL | 98 | -1.33 (0.22) |
|  |  |  | ExQW | 98 | -1.06 (0.22) |
|  |  |  | LIR | 646 | -1.16 (0.08) |
| **52 weeks** | | | | | |
| A12 | Dagogo | RCT | PLA/SC | 153 | 0.02 (0.09) |
|  |  |  | ERTU | 309 | -0.78 (0.11) |
| A43 | Rosenstock | RCT | PLA/SC | 139 | -0.54 (0.08) |
|  |  |  | DAPA | 281 | -1.10 (0.11) |
| A54 | Wilding | RCT | PLA/SC | 150 | 0.01 (0.08) |
|  |  |  | CANA | 307 | -0.85 (0.12) |
| A55 | Wilding | RCT | PLA/SC | 193 | -0.47 (0.10) |
|  |  |  | DAPA | 405 | -0.98 (0.14) |
| C1 | Ahmann | RCT | ExQW | 405 | -0.92 (0.06) |
|  |  |  | SEM | 404 | -1.55 (0.06) |
| C2 | Jabbour | RCT | DAPA | 230 | -1.23 (0.10) |
|  |  |  | ExQW | 227 | -1.38 (0.10) |
| C3 | Matthaei | RCT | PLA/SC | 108 | -0.10 (0.10) |
|  |  |  | DAPA | 108 | -0.80 (0.10) |
| C4 | Nauck | RCT | PLA/SC | 99 | 0.15 (0.10) |
|  |  |  | ALB | 197 | -0.79 (0.14) |
| C5 | Neal | RCT | PLA/SC | 690 | 0.03 (0.04) |
|  |  |  | CANA | 1382 | -0.62 (0.06) |
| C6 | Reusch | RCT | PLA/SC | 149 | -0.05 (0.07) |
|  |  |  | ALB | 149 | -0.80 (0.07) |
| C7 | Roden | RCT | PLA/SC | 228 | 0.09 (0.05) |
|  |  |  | EMPA | 448 | -0.76 (0.07) |
| D1 | Faher | OBS | LIX | 578 | Reference |
|  |  |  | LIR | 1736 | 0.30 (0.14) |
| D2 | McAdam-Marx | OBS | ExQW | 808 | -0.37 (0.08) |
|  |  |  | LIR | 4333 | -0.37 (0.09) |
| B6 | Jassim | OBS | ExBID | 114 | 0.08 (0.19) |
|  |  |  | LIR | 98 | 1.40 (1.60) |
| B7 | Mody | OBS | DUL | 585 | -3.13 (0.07) |
|  |  |  | LIR | 585 | -2.92 (0.07) |
| B7 | Mody | OBS | DUL | 422 | -3.24 (0.09) |
|  |  |  | ExQW | 422 | -2.92 (0.09) |
| NA | Hussein | OBS | CANA | 89 | -1.01 (0.19) |
|  |  |  | DAPA | 408 | -1.19 (0.09) |
|  |  |  | EMPA | 149 | -1.26 (0.15) |
|  |  |  | ExBID | 69 | -0.76 (0.24) |
|  |  |  | LIX | 109 | -0.54 (0.20) |
|  |  |  | DUL | 53 | -1.02 (0.30) |
|  |  |  | ExQW | 58 | -0.90 (0.27) |
|  |  |  | LIR | 416 | -1.01 (0.11) |
| *Abbreviations:* *PLA/SC, Placebo/Standard Care; CANA, Canagliflozin; DAPA, Dapagliflozin; EMPA, Empagliflozin; ERTU, Ertugliflozin; ExBID, Exenatide Twice Daily; LIX, Lixisenatide; ALB, Albiglutide; DUL, Dulaglutide; ExQW, Exenatide Once Weekly; LIR, Liraglutide; SEM, Semaglutide; TAS, Taspoglutide; OBS, Observational; RCT, Randomised Controlled Trial.* | | | | | |
